# The Inflammatory Bowel Disease—Disk Tool for Assessing Disability in Inflammatory Bowel Disease Patients: Validation of the Greek Version

**DOI:** 10.3390/jcm12083023

**Published:** 2023-04-21

**Authors:** Anastasia Katsoula, Georgios Axiaris, Afroditi Mpitouli, Maria Palatianou, Angeliki Christidou, Nikolaos Dimitriadis, Andreas Nakos, Ploutarchos Pastras, Panagiotis Kourkoulis, Pantelis Karatzas, Miltiadis Moutzoukis, Charalampos Zlatinoudis, Athanasios Philippidis, Anastasia Kourikou, Georgios Kokkotis, Antonios Gklavas, Angeliki Machaira, Aikaterini Mantaka, Persefoni Talimtzi, Evaggelia Anagnostopoulou, Ioannis E. Koutroubakis, Ioannis Papaconstantinou, Georgios Bamias, Spilios Manolakopoulos, Nicoletta Mathou, Konstantina Paraskeva, Andreas Protopappas, Eftychia Tsironi, Konstantinos H. Katsanos, Dimitrios K. Christodoulou, Georgios Papatheodoridis, Georgios Michalopoulos, Georgios Theocharis, Christos Triantos, Ioannis Pachiadakis, Konstantinos Soufleris, Nikolaos Viazis, Gerassimos J. Mantzaris, Georgios Tribonias, Maria Tzouvala, Angeliki Theodoropoulou, Konstantinos Karmiris, Evanthia Zampeli, Spyridon Michopoulos, Anna-Bettina Haidich, Olga Giouleme

**Affiliations:** 1 Department of Gastroenterology and Hepatology, 2nd Propedeutic Department of Internal Medicine, Medical School, Aristotle University, 54124 Thessaloniki, Greece; 2 Department of Gastroenterology, Alexandra General Hospital, 11528 Athens, Greece; 3 Department of Gastroenterology, Venizeleio General Hospital, 71409 Heraklion, Greece; 4 Department of Gastroenterology, General Hospital of Nikaia-Piraeus “Agios Panteleimon”, General Hospital Ditikis Attikis “Agia Varvara”, 12351 Athens, Greece; 5 Department of Gastroenterology, GHA ‘Evaggelismos—Polykliniki’, 10676 Athens, Greece; 6 Department of Gastroenterology, Theagenio Anticancer Hospital of Thessaloniki, 54639 Thessaloniki, Greece; 7 Department of Gastroenterology and Hepatology, 424 Military General Hospital, 54124 Thessaloniki, Greece; 8 GI-Unit, Department of Internal Medicine, University Hospital of Patras, 26504 Patras, Greece; 9 Department of Gastroenterology, Tzaneion General Hospital, 18536 Piraeus, Greece; 10 Department of Gastroenterology, Medical School of National and Kapodistrian University of Athens, General Hospital of Athens “Laiko”, 11527 Athina, Greece; 11 Department of Gastroenterology, University Hospital of Ioannina Medical School, 45500 Ioannina, Greece; 12 Department of Gastroenterology, Metaxa Memorial General Hospital, 18537 Piraeus, Greece; 13 Gastroenterology and Hepatology Division of the First Propedeutic Department of Internal Medicine, AHEPA University Hospital, 54636 Thessaloniki, Greece; 14 Gastroenterology-Liver Unit, Second Department of Internal Medicine, Hippokratio General Hospital, National and Kapodistrian University of Athens, 54643 Athens, Greece; 15 GI-Unit, 3rd Academic Department of Internal Medicine, Sotiria Hospital, National and Kapodistrian University of Athens, 11527 Athens, Greece; 16 Department of Surgery, Aretaieion University Hospital, 11528 Athens, Greece; 17 Department of Gastroenterology, University Hospital of Heraklion, 71500 Heraklion, Greece; 18 Department of Gastroenterology, General Hospital of Chania “Agios Georgios”, 73300 Chania, Greece; 19 Laboratory of Hygiene, Social—Preventive Medicine and Medical Statistics, Faculty of Medicine, School of Health Sciences, Aristotle University of Thessaloniki, 54124 Thessaloniki, Greece; 20 Department of Gastroenterology, “Konstantopoulio-Patission” General Hospital, 14233 Nea Ionia, Greece

**Keywords:** Crohn’s disease, ulcerative colitis, inflammatory bowel disease-disk, disability, validation

## Abstract

Background: The Inflammatory Bowel Disease-Disk (IBD-Disk) is a physician-administered tool that evaluates the functional status of patients with Inflammatory Bowel Disease (IBD). The aim of our study was to validate the content of the IBD-Disk in a Greek cohort of IBD patients. Methods: Two questionnaires [the IBD Disk and the IBD-Disability Index (IBD-DI)] were translated into Greek and administered to IBD patients at baseline visit, after 4 weeks and 6 months. Validation of the IBD Disk included measuring of concurrent validity, reproducibility, and internal consistency. Results: A total of 300 patients were included at baseline and 269 at follow-up. There was a good correlation between the total scores of the IBD-Disk and IBD-DI at baseline (Pearson correlation 0.87, *p* < 0.001). Reproducibility of the total IBD-Disk score was very good [intra-class correlation coefficient (ICC), 95% confidence interval (CI) 0.89 (0.86–0.91)]. Cronbach’s coefficient alpha for all items achieved 0.90 (95%CI 0.88–0.92), demonstrating a very good homogeneity of the IBD-Disk items. Female gender and extraintestinal manifestations were significantly associated with a higher IBD-Disk total score. Conclusions: The Greek version of the IBD-Disk proved to be a reliable and valid tool in detecting and assessing IBD-related disability in a Greek cohort of IBD patients.

## 1. Introduction

Inflammatory bowel diseases (IBD), comprising ulcerative colitis (UC) and Crohn’s disease (CD), are lifelong conditions with growing incidence and prevalence worldwide. IBD is characterized by a chronic relapsing—remitting, or continuously active course and may impact tremendously on patients’ personal, familial, social, and professional life affecting the overall quality of life (QoL). Thus, early onset of intensive treatment is often required to avoid complications, hospital admissions and/or surgical interventions to prevent irreversible bowel damage, IBD-related disability, maintain at least a near-to-normal QoL [1,2,3,4].

The ′IBD Disability Index’ (IBD-DI) was the first tool developed in collaboration with the World Health Organization (WHO) in an attempt to quantify disability. Disability is a patient-reported outcome that is defined by an objective limitation of function, and it is becoming a more important goal of IBD treatment. The IBD-DI is a physician-administered tool used to assess the functional status of IBD patients [5,6]. It is a 28-item validated questionnaire that covers all aspects of disability. A recent systematic review and meta-analysis concluded that the IBD-DI questionnaire is reliable and valid [7]. Although it provides a robust means of assessing IBD-related disability, it needs to be administered by a health care professional, is time-consuming, and thus mainly reserved for use in clinical trials. As a result, its complexity limits its use in routine clinical practice. To address this limitation, the IBD-Disk, a shortened, patient-friendly adaptation of the IBD-DI, was recently developed through a consensus-based process to provide a simple and quick method of assessing the level of disability experienced by patients with IBD [8]. The IBD-Disk is a 10-item visual instrument, exploring the general state of abdominal pain, body image, education and work, emotions, energy, interpersonal interactions, joint pain, defecation regulation, sexual functions, and sleep. Patients rate the previous ten items on an 11-point visual analogue scale ranging from 0 to 10, where 0 means “absolutely disagree” and 10 means “absolutely agree”, with an overall sum ranging from 0 to 100. The points on the disk are then connected to form a polygon. The area of the polygon can be interpreted as the size of the disease’s burden. As a result, this new tool provides the physician and patient with a visual representation of the disease burden at a given time, and it can also be used to demonstrate changes in disease burden over time by examining changes in the polygon.

The IBD-Disk has already been tested successfully in IBD patients in France and Portugal [9,10]. However, in different countries, patient acceptability, understanding, and response to self-administered tools such as IBD-disk are influenced by local socioeconomic factors, cultural trends, and medical systems [11,12]. As IBD behaves differently in ethnic groups, IBD-disk requires a cultural adaptation and translation process in other languages. Thus, in this study we aimed to develop a validated Greek version of the IBD-Disk.

## 2. Materials and Methods

### 2.1. Study Population and Design

This prospective multicenter cohort study was conducted in 19 Greek IBD referral centers. Patient recruitment was initiated on 1 September 2019, and ended on 31 July 2020, while patients’ follow-up was completed in October 2020. Adult patients with an established diagnosis of IBD were identified and recruited consecutively in the outpatient department of each center. Patients with an uncertain diagnosis, relevant psychiatric comorbidities that might influence disability, inability to complete the questionnaires, insufficient knowledge of Greek language, and refusal to participate were excluded.

This study was based on the COSMIN (COnsensus-based Standards for the selection of health Measurement INstruments) checklist, a validated tool developed to evaluate the methodological quality of studies, which explore measurement properties of patient reported outcomes and as guidance for designing them [13,14,15]. The protocol was approved by the research Ethics Committee at each participating center. The study was conducted under Good Clinical Practice Guidelines and the ethical principles of the Declaration of Helsinki.

### 2.2. Translation and Cross-Cultural Adaptation

After receiving authorization from the developers of the IBD-Disk, the original version was translated backward-forward in Greek as the instrument was developed in English (Table S1). This step included forward translation by two expert native Greek translators followed by backward translation by two expert English native translators with synthesis of the two versions after each step by an expert committee. Then the translated IBD-Disk was pretested by 5 IBD patients to check the items’ interpretation, ease of comprehension, and cultural relevance. No significant modification was needed as the questionnaire was noted as simple, clear, and easy to complete during the pretest.

### 2.3. Questionnaire Administration and Data Collection Procedures

After providing informed consent, patients were administered the questionnaire (IBD-Disk) by a health care provider. For validation purposes, patients were also asked to complete the IBD-DI questionnaire. Four weeks and 6 months after the initial visit, patients completed an identical IBD-Disk questionnaire. All questionnaires were self-completed by the patients. The following characteristics were collected at baseline and during subsequent visits (those modifiable): patients’ age, gender, smoking status, disease phenotype according to Montreal classification, prior IBD-related surgery, IBD-related medical therapy, extraintestinal manifestations and clinical disease activity assessed by the Harvey Bradshaw Index (HBI) for CD and the partial Mayo score for UC [16,17].

The IBD–Disk consists of 10 questions, exploring the general state of abdominal pain, interpersonal interactions, body image, emotions, energy, joint pain, defecation regulation, education and work, sexual functions, and sleep. The answers are marked on 11-point visual analogue scales, from 0 to 10, where 0 means “absolutely disagree” and 10 means “absolutely agree” with an overall sum ranging from 0 to 100. The total IBD-DI score was calculated using the following formula: overall sum × 100/(*p* × 4), in which *p* indicated the number of questions answered. A higher score was associated with a worse level of disability [5].

### 2.4. Statistical Analysis

The sample size was determined according to feasibility criteria, which suggests that a total of 10 subjects are needed per item for validation of the questionnaire [18]. Since the IBD-Disk questionnaire consists of 10 items, a sample size of 100 subjects was set as a minimum requirement. We increased the sample to 300 patients, expecting a high rate of loss to follow-up, as is usually observed in studies with similar design.

Continuous variables were presented with mean values ± standard deviation, whereas for categorical variables frequencies and percentages were used. To assess the concurrent validity of the total IBD-Disk score, Pearson’s correlation coefficient between the IBD-Disk and the IBD-DI total score was calculated. To assess the concurrent validity per item, box plot figures were created displaying the IBD-Disk scores per item (0–10) according to corresponding IBD-DI responses. Moreover, univariate linear regression analysis was used to compute regression coefficients (beta coefficients) and their 95% confidence intervals (95%CI). A regression coefficient significantly different from zero was considered as exhibiting good consistency between the IBD-Disk and IBD-DI items. To assess test-retest reliability, the intra-class correlation coefficient (ICC) and its 95%CI for each IBD-Disk item and the total score were calculated. An ICC value between 0.75–0.90 was considered as good and a value above 0.90 as excellent [19]. Internal consistency of the IBD-Disk was assessed using Cronbach’s coefficient alpha between combinations of items. A value between 0.70–0.80 was considered as acceptable, 0.80–0.90 as good and above 0.90 as excellent [20]. 

Linear regression analysis was used for the association of clinical factors with the IBD-Disk total score at baseline in the total sample. Factors with a *p*-value < 0.2 in the univariate analysis were entered into a multivariate regression model. The same analysis was run separately for patients with CD and UC.

Change in the IBD-Disk total score between baseline and follow-up visits according to disease activity was assessed using paired *t*-test. Disease was defined as active if the HBI score was > 4 or partial Mayo score ≥ 2. Four groups of patients were formed according to disease activity: patients inactive at both visits, active at baseline becoming inactive at follow-up, active at both visits, inactive at baseline becoming active at follow-up. Differences among these groups were tested in the total sample and separately in patients with CD and UC, using Chi-squared test.

Logistic regression analysis was used for the association of any change in different IBD-Disk items with disease relapse. Disease relapse was defined as if IBD was inactive at baseline visit and became active at follow-up. Items with a *p*-value < 0.2 in the univariate analysis were entered into a multivariate regression model. The same analysis was run separately for patients with CD and UC.

Statistical analysis was performed using IBM SPSS software (IBM Corp. Released 2019. IBM SPSS Statistics for Windows, Version 26.0. Armonk, NY: IBM Corp, New York, NY, USA) and the significance level was set at α = 0.05.

## 3. Results

### 3.1. Patient Characteristics

In total, 370 patients were initially screened for inclusion in the study. Seventy patients were excluded due to at least one missing value in one of the two questionnaires. Thus, the population under evaluation consisted of 300 patients (199 patients diagnosed with CD, 101 with UC) at baseline visit and of 269 (177 patients diagnosed with CD, 92 with UC) at both follow-up visits. Clinical and demographic characteristics are reported in Table 1. At baseline, the mean IBD-Disk score was 27.24 ± 22.76, and the mean IBD-DI score was 25.68 ± 19.33. The mean age was 41.3 ± 13.7 years and 161 patients (53.7%) were males. One hundred thirty-five CD patients (69.6%) and 72 UC patients (72.0%) were in clinical remission at baseline visit. Medications for IBD at baseline included aminosalicylates in 96 (32%), steroids in 39 (13%), immunosuppressants in 85 (28.3%) and biologics in 237 (79%) patients, respectively. In total, 36 patients (12%) had undergone at least one IBD-related surgery. Extraintestinal manifestations were reported in 120 patients (40%).

### 3.2. Validity of the IBD-Disk Score

**Figure 1:**
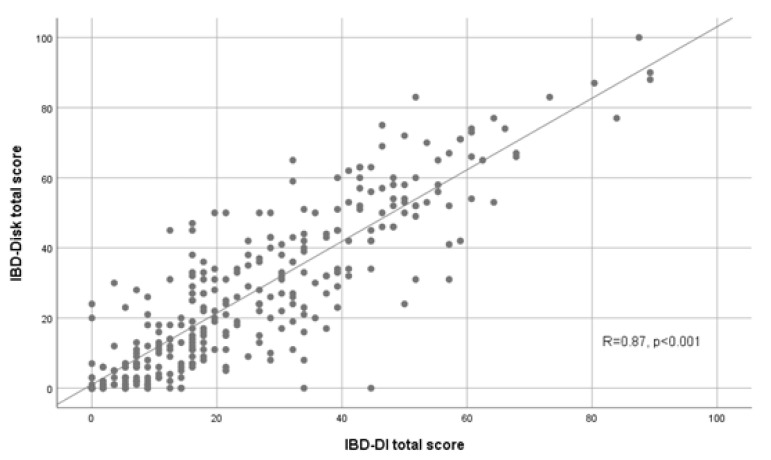
Correlation between the IBD-Disk total score and IBD-DI total score at baseline (*n* = 300).

**Figure 2:**
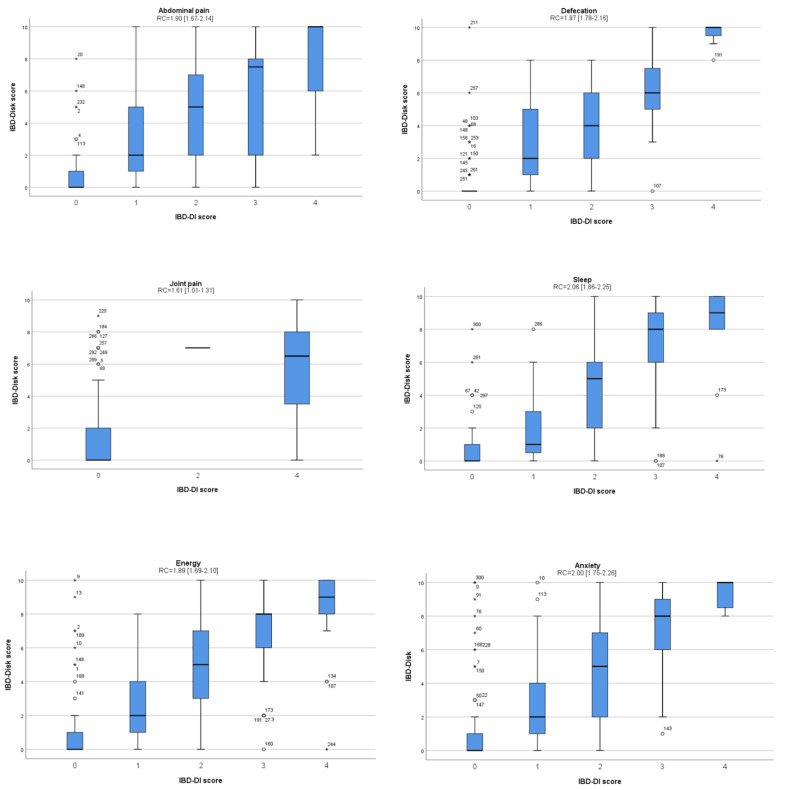

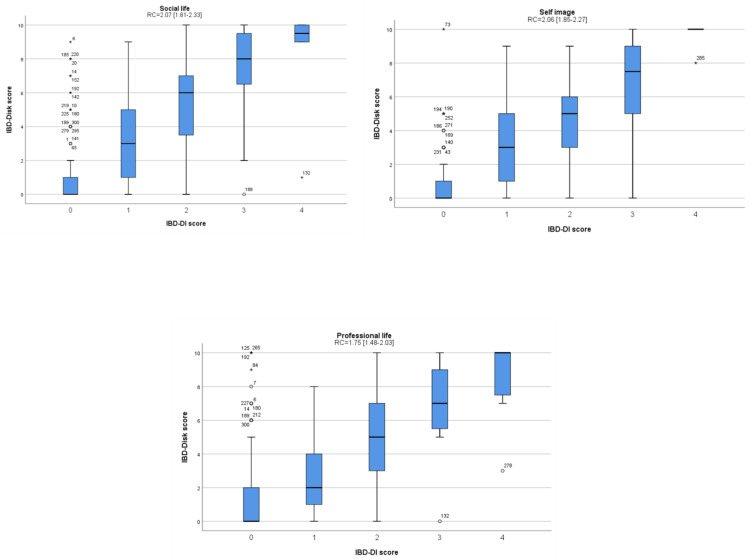
Reliability of the IBD-Disk compared to the IBD-DI per item at baseline (*n* = 300).

Validity examines whether the instrument measures the outcome of interest. There was a good correlation between the total scores of IBD-Disk and IBD-DI at baseline, with a Pearson correlation of 0.87 (*p* < 0.001) (Figure 1). Regarding the validity per item at baseline, medians of IBD-Disk scores had a stepped appearance from the lowest to the highest values between the IBD-DI responses groups (Figure 2). All associated linear regression coefficients were significantly different from 0. These results suggest a good consistency between the IBD-Disk items and the corresponding questions in the IBD-DI.

### 3.3. Reliability of the IBD-Disk Score

Reproducibility of the total IBD-Disk score was good (ICC [95%CI] = 0.89 [0.86–0.91]). All the items included in the IBD-Disk had an ICC value between 0.76 and 0.90 which indicate a good reproducibility (Table 2).

Cronbach’s coefficient alpha was positive for each pair of the IBD-Disk items at baseline. The pairs of items which showed the best correlation were [abdominal pain + defecation], [social life + defecation], [social life + professional life], [energy + professional life], [energy + sleep], [social life + anxiety], [professional life + anxiety], [energy + anxiety] and [social life + sexual function]. On the other hand, the pairs of items which were the least interconnected were [self-image + abdominal pain], [self-image + defecation] and [self-image + social life]. Cronbach’s coefficient alpha for all items achieved 0.90 [95%CI = 0.88–0.92], demonstrating a near to excellent homogeneity of the IBD-Disk items (Table 3).

### 3.4. Analysis of Clinical Factors Associated with the IBD-Disk Score

Gender and extraintestinal manifestations were significantly associated with the IBD-Disk total score. Specifically, female patients had an increased mean IBD-Disk score by 12.66 units compared to male patients, adjusting for type of the disease and extraintestinal manifestations. Patients with extraintestinal manifestations had also an increased mean IBD-Disk total score by 11.74 units compared to those without, adjusting for type of the disease and gender (Table S2). IBD type, age, disease duration, disease location and history of IBD-related surgery did not influence the IBD-Disk score.

In CD patients, gender and extraintestinal manifestations were also significantly associated with the IBD-Disk total score. Specifically, female patients had an increased mean IBD-Disk score by 13.25 units compared to male patients, adjusting for disease behavior, disease location and extraintestinal manifestations. Patients with extraintestinal manifestations had an increased mean IBD-Disk total score by 13.32 units compared to those without, adjusting for gender, disease behavior and disease location (Table S3).

In UC patients, only extraintestinal manifestations were significantly associated with the IBD-Disk total score. Specifically, patients with extraintestinal manifestations had an increased mean IBD-Disk total score by 9.58 units compared to those without, adjusting for gender (Table S4).

### 3.5. IBD-Disk Score Variability between Baseline and Follow-up Visits

The median IBD-Disk total score significantly decreased in patients who were active at baseline and became inactive at follow-up (*p* = 0.003), while it significantly increased in patients who were inactive at baseline and became active at follow-up (*p* = 0.025) (Table 4).Two-third of cases (58%) were inactive at both visits, 16% were active at both visits, 15% were active at baseline and became inactive at follow-up, and 11% were inactive at baseline and became active at follow-up.

In univariate analysis, it was shown that, for one-unit decrease in the abdominal pain score from baseline to follow-up, the odds for a relapse decreased by 15%. For one-unit decrease in the professional life score from baseline to follow-up, the odds for a relapse decreased by 14%. For one-unit decrease in change in the energy score from baseline to follow-up, the odds for a relapse decreased by 13% (Table S5). However, in a multivariate analysis, none of these changes seemed to significantly affect the relapse

In CD patients, in univariate analysis, for one-unit decrease in the abdominal pain score from baseline to follow-up, the odds for a relapse decreased by 17% (Table S6). In UC patients, none of the changes from baseline to follow-up visits in the individual IBD-Disk items seemed to significantly affect a relapse (Table S7).

## 4. Discussion

In a prospective multicentre study, we evaluated the newly introduced, self-administered IBD disk in a Greek IBD population. Our data showed that the IBD-Disk is a valid and reliable instrument for measuring disability, with high internal consistency and test-retest reliability. We compared it with the already validated IBD-DI and the IBD-Disk items correlated well with the corresponding IBD-DI questions. The IBD-Disk total score elevated with increased disease activity. Moreover, female patients had higher disability scores, irrespective of other disease characteristics. Finally, the presence of extraintestinal manifestations seems to be associated with increased disability in both UC and CD patients. Our findings confirmed and reinforced evidence from previous validation studies in French and Portugese patients [9,10]. Since self-administered tools like IBD-disk are influenced by the local socio-cultural sensitivities and practices, validation studies in different populations are necessary for universal application of IBD-disk [11,12]. Therefore, we carried out a valid translation process and required cultural adaptations based on COSMIN recommendations, producing a Greek translation of the IBD-Disk questionnaire. Subsequently, we applied the questionnaire in both CD and UC patients, producing results comparable to French study, that promote the dissemination of IBD disk to other countries.

The IBD-Disk total score was positively correlated with disease activity. Patients with clinically active UC or CD had higher disability scores. In addition, contrary to French study, in our cohort, the remission or the deterioration of the disease during follow-up was followed by change in IBD-Disk score, accordingly. These findings confirmed previous research which showed that clinical activity of the disease is a significant component of IBD-related disability [9,21]. Therefore, IBD-Disk may help in early detection of disease relapse.

Furthermore, we found that patients with extraintestinal manifestations and female gender had a significantly higher IBD-Disk total score. Interestingly, we found no relation between the IBD-Disk score and IBD type, phenotype, location, and history of IBD-related surgery. Gender-based differences in IBD pathogenesis and disease course are multifactorial and complex including both genetic predisposition and environmental exposures Several studies have shown that females have lower self-reported QoL aspects than males, which is consistent with our findings [22,23,24]. The IBD-Disk score was also associated with gender in a French validation study involving 447 IBD patients [9]. Similarly, a Portuguese cohort found that females had significantly higher IBD-Disk scores [10]. A large multi-center study including 1700 patients revealed that female patients had a significantly higher rate of moderate to severe disability [25]. Several explanations, including the effect of gender hormones, gender-dependent genetic alterations, and gender-associated compositional diversion in the gut microbiome, may underlie such gender differences in disability among patients. More research is needed, however, to identify mediating factors that might impact on the disability between genders. Previous research has found that the presence of extraintestinal manifestations is associated with increased disability in both UC and CD patients, which our study confirmed [25,26,27]. This finding could be explained by the fact that most individuals with extraintestinal manifestations have active disease, a condition associated with disability.

This study’s main strength is its sample. The sample size was large, and it should be noted that all IBD patients in Greece have open and easy access to IBD referral centers, allowing patients with mild disease to be recruited as well. Indeed, the recruitment of patients in our study from general population provided the full spectrum of disability necessary to validate the IBD-Disk. At the baseline visit, three hundred patients were included, and 269 of them responded to the questionnaires at the follow-up. The amount of missing data for each of the ten items was very low, with only 11% of patients failing to respond to the questionnaires despite their presence at the follow-up visit, demonstrating the questionnaires’ acceptability [9,28].

Another strength of our study comes from the methods used to validate the IBD-Disk. We used a validated methodology based on the COSMIN checklist, including all steps of quantitative validation [13,14,15]. 

Nevertheless, there are certain limitations in our study. The patient population was not quite diverse. The majority of study participants were recruited from tertiary referral centers, which may have resulted in a recruitment bias. As a result, the study population may not be representative of the general IBD population. Indeed, 66% of the patients included had CD and more than two thirds were treated with biological agents.

In conclusion, to our knowledge, this is the first attempt to evaluate the use of the IBD Disk for quantifying disability in a Greek cohort of IBD patients. The IBD-Disk has been proved to be a valid and reliable instrument for assessing IBD-related disability in culturally diverse populations. Further research with prospective studies is required, as the IBD-Disk may also have an additive value in the long-term monitoring of IBD-related disability.

## Figures and Tables

**Table 1:** Clinical characteristics of the study participants at baseline (*n* = 300).

	Total (*n* = 300)
Gender, *n* (%)	
Male	161 (53.7)
Female	139 (46.3)
Age (years), mean (SD)	41.3 (13.7)
Disease type, *n* (%)	
Crohn’s disease	199 (66.3)
Ulcerative colitis	101 (33.7)
Disease duration (years), *n* (%)	
<2	24 (8)
2–8	139 (46.3)
>8	137 (45.7)
Age at diagnosis (years, CD patients), *n* (%)	
A1	34 (17.1)
A2	123 (61.8)
A3	42 (21.1)
Disease behavior (CD patients), *n* (%)	
B1	114 (57.3)
B2	51 (25.6)
B3	34 (17.1)
Perianal involvement, *n* (%)	44 (22.1)
Disease location in CD, *n* (%)	
Ileal [L1]	76 (38.2)
Colonic [L2]	24 (12.1)
Ileocolonic [L3]	99 (49.7)
Upper GI involvement [L4]	13 (6.5)
Disease location in UC, *n* (%)	
Proctitis [E1]	9 (8.9)
Left-sided colitis [E2]	40 (39.6)
Pancolitis [E3]	52 (51.5)
Extraintestinal manifestations, *n* (%)	120 (40.0)
Biologics/small molecules, *n* (%)	
None	63 (21.0)
Infliximab	127 (42.3)
Adalimumab	30 (10.0)
Ustekinumab	25 (8.3)
Vedolizumab	46 (15.3)
Golimumab	6 (2.0)
Tofacitinib	2 (0.7)
Cyclosporin	1 (0.3)
AZA, *n* (%)	72 (24.0)
MTX, *n* (%)	13 (4.3)
5-ASA, *n* (%)	96 (32.0)
Steroids, *n* (%)	39 (13.0)
Smoking, *n* (%)	79 (26.3)
IBD-related surgery, *n* (%)	36 (12.0)
Hospitalization, *n* (%)	40 (13.3)
pMayo, *n* (%)	
pMayo < 2	73 (72.2)
pMayo 2–5	25 (24.8)
pMayo > 5	3 (3.0)
HBI, *n* (%)	
HBI < 4	138 (69.3)
HBI 4–12	57 (28.6)
HBI > 12	4 (2.1)

SD, standard deviation; CD, Crohn’s disease; GI, gastrointestinal; UC, ulcerative colitis; AZA, Azathioprine; MTX, Methotrexate; 5-ASA, 5-aminosalicylates; IBD, Inflammatory bowel disease; pMayo, Partial Mayo Score; HBI, Harvey-Bradshaw Index.

**Table 2:** “Test-retest” reliability of the IBD-Disk: Intraclass correlation coefficients for each item and the total IBD-Disk score (*n* = 300).

	ICC [95%CI]
Abdominal pain	0.78 [0.73–0.83]
Defecation	0.76 [0.70–0.81]
Social life	0.85 [0.81–0.88]
Professional life	0.79 [0.74–0.84]
Sleep	0.79 [0.74–0.83]
Energy	0.82 [0.78–0.86]
Anxiety	0.79 [0.74–0.84]
Self-image	0.88 [0.85–0.91]
Sexual function	0.82 [0.77–0.85]
Joint pain	0.87 [0.83–0.89]
Total score	0.89 [0.86–0.91]

**Table 3:** Internal consistency reliability (Cronbach’s coefficient alpha) for each pair of the IBD-Disk items at baseline (*n* = 300).

	Abdominal Pain	Defecation	Social Life	Professional Life	Sleep	Energy	Anxiety	Self-Image	Sexual Function	Joint Pain
Abdominal pain	-									
Defecation	0.75	-								
Social life	0.65	0.77	-							
Professional life	0.68	0.72	0.89	-						
Sleep	0.55	0.53	0.66	0.72	-					
Energy	0.71	0.63	0.70	0.78	0.76	-				
Anxiety	0.61	0.55	0.77	0.74	0.69	0.80	-			
Self-image	0.40	0.31	0.48	0.52	0.56	0.56	0.52	-		
Sexual function	0.54	0.53	0.78	0.70	0.61	0.65	0.72	0.61	-	
Joint pain	0.64	0.62	0.58	0.66	0.63	0.68	0.59	0.55	0.61	-

**Table 4:** Change in the IBD-Disk total score between baseline and follow-up (*n* = 269).

IBD-Disk Total Score, Median (IQR)															
Inactive at Both Visits ^a^				Active Becoming Inactive ^a^				Active at Both Visits ^a^				Inactive Becoming Active ^a^			
	B	FU	*p*-Value		B	FU	*p*-Value		B	FU	*p*-Value		B	FU	*p*-Value
Total (*n* = 157)	13 (3, 30.5)	16 (4, 29.5)	0.364	Total (*n* = 39)	32 (13, 52)	21 (9, 43)	0.003 *	Total (*n* = 43)	53 (31, 67)	46 (33, 59)	0.260	Total (*n* = 30)	25.5 (11, 37.5)	39 (12.5, 45.2)	0.025 *
CD (*n* = 107)	13 (3, 31)	17 (4, 30)	0.364	CD (*n* = 27)	34 (19, 59)	23 (11, 43)	0.005 *	CD (*n* = 28)	54 (33.5, 72.2)	49.5 (36, 62.2)	0.062	CD (*n* = 15)	24 (13, 42)	40 (13, 53)	0.096
UC (*n* = 50)	15.5 (4.5, 28.2)	12.5 (4, 28.2)	0.779	UC (*n* = 12)	23.5 (7, 43.5)	11 (7.3, 41)	0.286	UC (*n* = 15)	42 (12, 60)	46 (19, 54)	0.209	UC (*n* = 15)	31 (3, 36)	33 (10, 42)	0.140

IQR: Interquartile range; B: Baseline; FU: Follow-up; CD: Crohn’s disease; UC: Ulcerative colitis. ^a^ Definition of disease activity: active if HBI score ≥ 4 (CD) or P-MAYO score ≥ 2 (UC). * Statistically significant at level 0.05.

## Data Availability

Data is unavailable due to privacy or ethical restrictions.

## Supplementary Materials

The following supporting information can be downloaded at: https://www.mdpi.com/article/10.3390/jcm12083023/s1, Table S1: The table presents questions from the original English version of the IBD Disk and its final Greek translation; Table S2: Linear regression analysis for the association of clinical factors with the IBD-Disk total score (n = 300); Table S3: Linear regression analysis for the association of clinical factors with IBD-Disk total score in patients with Crohn’s disease (n = 199); Table S4: Linear regression analysis for the association of clinical factors with IBD-Disk total score in patients with Ulcerative colitis (n = 101); Table S5: Univariate analysis for disease outbreak (events = 30) from baseline to follow-up (n = 269); Table S6: Univariate analysis for disease outbreak (events = 15) from baseline to follow-up in CD patients (n = 199); Table S7: Univariate analysis for disease outbreak (events = 15) from baseline to follow-up in UC patients (n = 101).

## References

[1] Moradkhani A., Beckman L.J., Tabibian J.H. (2013). Health-related quality of life in inflammatory bowel disease: Psychosocial, clinical, socioeconomic, and demographic predictors. J. Crohn’s Col..

[2] Shafer L.A., Walker J.R., Restall G., Chhibba T., Ivekovic M., Singh H., Targownik L.E., Bernstein C.N. (2019). Association Between IBD Disability and Reduced Work Productivity (Presenteeism): A Population-Based Study in Manitoba, Canada. Inflamm. Bowel Dis..

[3] Marín L., Mañosa M., Garcia-Planella E., Gordillo J., Zabana Y., Cabré E., Domènech E. (2013). Sexual function and patients’ perceptions in inflammatory bowel disease: A case–control survey. J. Gastroenterol..

[4] McDermott E., Mullen G., Moloney J., Keegan D., Byrne K., Doherty G.A., Cullen G., Malone K., Mulcahy H.E. (2015). Body Image Dissatisfaction: Clinical Features, and Psychosocial Disability in Inflammatory Bowel Disease. Inflamm. Bowel Dis..

[5] Gower-Rousseau C., Sarter H., Savoye G., Tavernier N., Fumery M., Sandborn W.J., Feagan B.G., Duhamel A., Guillon-Dellac N., Colombel J.F. (2017). Validation of the Inflammatory Bowel Disease Disability Index in a population-based cohort. Gut.

[6] Peyrin-Biroulet L., Cieza A., Sandborn W.J., Coenen M., Chowers Y., Hibi T., Kostanjsek N., Stucki G., Colombel J.F. (2012). Development of the first disability index for inflammatory bowel disease based on the international classification of functioning, disability and health. Gut.

[7] Lo B., Prosberg M.V., Gluud L.L., Chan W. (2018). Systematic review and meta-analysis: Assessment of factors affecting disability in inflammatory bowel disease and the reliability of the inflammatory bowel disease disability index. Alim. Pharmacol. Ther..

[8] Ghosh S., Louis E., Beaugerie L., Bossuyt P., Bouguen G., Bourreille A., Ferrante M., Franchimont D., Frost K., Hebuterne X. (2017). Development of the IBD Disk: A Visual Self-administered Tool for Assessing Disability in Inflammatory Bowel Diseases. Inflamm. Bowel Dis..

[9] Le Berre C., Flamant M., Bouguen G., Siproudhis L., Dewitte M., Dib N., Cesbron-Metivier E., Goronflot T., Hanf M., Gourraud P.A. (2020). VALIDation of the IBD-Disk Instrument for Assessing Disability in Inflammatory Bowel Diseases in a French Cohort: The VALIDate Study. J. Crohn’s Col..

[10] Mendes S.S., Ferreira P., Antunes P., Gonçalves M., Leal T., Gonçalves B., Rebelo A., Arroja B., Caetano A.C., Gonçalves R. (2021). Validation of the IBD-Disk in a Portuguese cohort. Eur. J. Gastroenterol. Hepatol..

[11] Shi H.Y., Levy A.N., Trivedi H.D., Chan F., Ng S.C., Ananthakrishnan A.N. (2018). Ethnicity influences phenotype and outcomes in inflammatory bowel disease: A systematic review and meta-analysis of population-based studies. Clin. Gastroenterol. Hepatol..

[12] Agrawal M., Cohen-Mekelburg S., Kayal M., Axelrad J., Galati J., Tricomi B., Kamal K., Faye A.S., Abrudescu P., Scherl E. (2019). Disability in inflammatory bowel disease patients is associated with race, ethnicity and socio-economic factors. Aliment. Pharmacol. Ther..

[13] Mokkink L.B., Prinsen C., Patrick D.L., Alonso J., Bouter L., de Vet H.C., Terwee C.B., Mokkink L. (2018). COSMIN guideline for systematic reviews of patient-reported outcome measures. Qual. Life Res..

[14] Mokkink L.B., De Vet H.C., Prinsen C.A., Patrick D.L., Alonso J., Bouter L.M., Terwee C.B. (2018). COSMIN Risk of Bias checklist for systematic reviews of Patient-Reported Outcome Measures. Qual. Life Res. Int. J. Qual. Life Aspects Treat. Care Rehab..

[15] Terwee C.B., Prinsen C.A., Chiarotto A., Westerman M.J., Patrick D.L., Alonso J., Bouter L.M., De Vet H.C., Mokkink L.B. (2018). COSMIN methodology for evaluating the content validity of patient-reported outcome measures: A Delphi study. Qual. Life Res. Int. J. Qual. Life Aspects Treat. Care Rehab..

[16] Harvey R.F., Bradshaw J.M. (1980). A simple index of Crohn’s-disease activity. Lancet.

[17] Lewis J.D., Chuai S., Nessel L., Lichtenstein G.R., Aberra F.N., Ellenberg J.H. (2008). Use of the noninvasive components of the Mayo score to assess clinical response in ulcerative colitis. Inflamm. Bowel Dis..

[18] Streiner D.N.G. (1989). Health Measurement Scales.

[19] Shrout P.E., Fleiss J.L. (1979). Intraclass correlations: Uses in assessing rater reliability. Psychol. Bull..

[20] Bolarinwa O.A. (2015). Principles and methods of validity and reliability testing of questionnaires used in social and health science researches. Niger. Postgrad. Med. J..

[21] Allen P.B., Gower-Rousseau C., Danese S., Peyrin-Biroulet L. (2017). Preventing disability in inflammatory bowel disease. Ther. Adv. Gastroenterol..

[22] Nurmi E., Haapamäki J., Paavilainen E., Rantanen A., Hillilä M., Arkkila P. (2013). The burden of inflammatory bowel disease on health care utilization and quality of life. Scand. J. Gastroenterol..

[23] Greuter T., Manser C., Pittet V., Vavricka S.R., Biedermann L. (2020). Gender Differences in Inflammatory Bowel Disease. Digestion.

[24] Rustgi S.D., Kayal M. (2020). Sex-based differences in inflammatory bowel diseases: A review. Ther. Adv. Gastroenterol..

[25] Tannoury J., Nachury M., Martins C., Serrero M., Filippi J., Roblin X., Bourrier A., Bouguen G., Franchimont D., Savoye G. (2021). Determinants of IBD-related disability: A cross-sectional survey from the GETAID. Alim. Pharmacol. Ther..

[26] Lo B., Julsgaard M., Vester-Andersen M.K., Vind I., Burisch J. (2018). Disease activity, steroid use and extraintestinal manifestation are associated with increased disability in patients with inflammatory bowel disease using the inflammatory bowel disease disability index: A cross-sectional multicentre cohort study. Eur. J. Gastroenterol. Hepatol..

[27] Marinelli C., Savarino E. (2019). Factors Influencing Disability and Quality of Life during Treatment: A Cross-Sectional Study on IBD Patients. Gastroenterol. Res. Pract..

[28] Yoon J.Y., Shin J.E., Park S.H., Park D.I., Cha J.M. (2017). Disability due to Inflammatory Bowel Disease Is Correlated with Drug Compliance, Disease Activity, and Quality of Life. Gut Liver.

