# Supplementary material for: The Inflammatory Bowel Disease—Disk Tool for Assessing Disability in Inflammatory Bowel Disease Patients: Validation of the Greek Version
Source: J Clin Med. 2023 Apr 21;12(8):3023. doi: 10.3390/jcm12083023 (PMC10143768; doi:10.3390/jcm12083023)
Supplement: Supplementary file 1 [file jcm-12-03023-s001.zip › jcm-2204512-supplementary.pdf]

Table S1: The table presents questions from the original English version of the IBD Disk and its final Greek translation

**English version**		**Ελληνική εκδοχή**	
In the last week, because of my Crohn's disease or ulcerative colitis:		Την τελευταία εβδομάδα εξαιτίας της νόσου Crohn ή της ελκώδους κολίτιδας…	
Abdominal pain	…I have had aches or pains in my stomach or abdomen	Κοιλιακό άλγος	…Είχα πόνους στο στομάχι ή στην κοιλιά
Regulating defecation	…I have had difficulty coordinating and managing defecation, including choosing and getting to an appropriate place for defecation and cleaning myself afterwards	Έλεγχος κενώσεων	…Είχα δυσκολία στο να συντονίσω και να διαχειριστώ τις κενώσεις μου, όπως επίσης και στο να διαλέξω και να φτάσω σε ένα κατάλληλο μέρος για κένωση, καθώς και στο να καθαρίσω τον εαυτό μου μετά
Interpersonal interactions	…I have had difficulty with personal relationships and/or difficulty participating in the community	Διαπροσωπικές σχέσεις	…Είχα δυσκολία στις προσωπικές μου σχέσεις και/ή δυσκολία συμμετοχής στο κοινωνικό περιβάλλον
Education and work	…I have had difficulty with school or studying activities, and/or difficulty with work or household activities	Εκπαίδευση και εργασία	…Είχα δυσκολία στο σχολείο ή στις εκπαιδευτικές δραστηριότητες, και/ή δυσκολία στην εργασία ή στο νοικοκυριό
Sleep	…I have had difficulty sleeping, such as falling asleep, waking up frequently during the night or waking up too early in the morning	Ύπνος	…Είχα δυσκολία στον ύπνο, όπως στο να αποκοιμηθώ, σηκωνόμουν πολλές φορές κατά τη διάρκεια της νύχτας ή ξυπνούσα πολύ νωρίς το πρωί
Energy	…I have not felt rested and refreshed during the day, and have felt tired and without energy	Ενεργητικότητα	…Δεν έχω νιώσει ξεκούραστος ή ανανεωμένος κατά τη διάρκεια της ημέρας, και έχω νιώσει κουρασμένος και χωρίς ενέργεια
Emotions	…I have felt sad, low or depressed, and/ or worried or anxious	Συναισθήματα	…Έχω νιώσει λυπημένος, «πεσμένος» ή καταθλιπτικός, και/ή ανήσυχος ή αγχωμένος
Body image	…I have not liked the way my body or body parts look	Εικόνα σώματος	…Δεν μου αρέσει το πως φαίνεται το σώμα μου ή σημεία του σώματός μου
Sexual functions	…I have had difficulty with the mental and/or physical aspects of sex	Σεξουαλική λειτουργικότητα	…Είχα δυσκολία με τις ψυχικές και/ή τις σωματικές πτυχές της σεξουαλικής επαφής
Joint pain	…I have had pains in the joints of my body	Άλγος αρθρώσεων	…Είχα πόνους στις αρθρώσεις του σώματός μου

Table S2: Linear regression analysis for the association of clinical factors with the IBD-Disk total score (n=300)

	**Univariate analysis**			**Multivariate analysis**		
	**Coefficient**	**95%CI**	**p-value**	**Coefficient**	**95%CI**	**p-value**
**Gender**						
Male	Ref.			Ref.		**<0.001***
Female	14.87	9.95, 19.78	**<0.001***	12.66	7.83, 17.48	
**Age (years)**	-0.04	-0.23, 0.15	0.681			
**Disease**						
Crohn's disease	Ref.			Ref.		0.289
Ulcerative colitis	-4.18	-9.65, 1.28	**0.133**	-2.71	-7.73, 2.31	
**Disease duration(years)**	0.16	-0.12, 0.45	0.252			
**Extraintestinal manifestations**						
No	Ref.					**<0.001***
Yes	14.29	9.26, 19.33	**<0.001***	11.74	6.82, 16.66	
**Surgery**						
No	Ref.					
Yes	-1.39	-9.37, 6.58	0.731			

*Statistically significant at level 0.05

Table S3: Linear regression analysis for the association of clinical factors with IBD-Disk total score in patients with Crohn's disease (n=199)

	**Univariate analysis**			**Multivariate analysis**		
	**Coefficient**	**95%CI**	**p-value**	**Coefficient**	**95%CI**	**p-value**
**Gender**						
Male	Ref.			Ref.		
Female	19.32	13.26, 25.39	**<0.001***	13.25	6.06, 20.44	**<0.001***
**Age (years)**	-0.01	-0.26, 0.25	0.962			
**Disease duration(years)**	0.15	-0.29, 0.58	0.499			
**Disease behavior**						
B1	Ref.			Ref.		
B2	-3.33	-12.05, 5.37	**0.452**	-3.43	-11.26, 4.40	0.388
B3	-9.07	-19.04, 0.91	**0.075**	-8.52	-17.67, 0.64	0.068
**Disease location**						
L1	Ref.			Ref.		
L2	10.57	-1.16, 22.29	**0.077**	8.01	-3.42, 19.43	0.168
L3	-0.38	-7.71, 6.96	**0.920**	-0.35	-7.69, 6.99	0.925
L4	8.41	-5.58, 22.39	**0.237**	10.97	-3.11, 25.06	0.126
**Extraintestinal manifestations**						
No	Ref.					
Yes	16.09	9.78, 22.41	**<0.001***	13.32	6.40, 20.23	**<0.001***
**Surgery**						
No	Ref.					
Yes	-3.59	-11.31, 6.12	0.557			

*Statistically significant at level 0.05

Table S4: Linear regression analysis for the association of clinical factors with IBD-Disk total score in patients with Ulcerative colitis(n=101)

	Univariate analysis			Multivariate analysis		
	**Coefficient**	**95%CI**	**p-value**	**Coefficient**	**95%CI**	**p-value**
**Gender**						
Male	Ref.			Ref.		
Female	5.56	-2.65, 13.76	0.182	5.27	-2.78, 13.31	0.197
**Age (years)**	-0.06	-0.35, 0.23	0.682			
**Disease duration(years)**	0.22	-0.14, 0.57	0.233			
**Disease distribution**						
L2	Ref.					
L3	-6.31	-31.61, 22.29	0.621			
E1	-0.61	-26.99, 25.78	0.964			
E2	9.18	-13.23, 31.59	0.417			
E3	5.96	-16.17, 28.09	0.594			
**Extraintestinal manifestations**						
No	Ref.			Ref.		
Yes	9.75	1.35, 18.16	**0.023***	9.58	1.20, 17.96	**0.025***
**Surgery**						
No	Ref.					
Yes	-17.63	-58.93, 23.67	0.399			
*Statistically significant at level 0.05						

Table S5: Univariate analysis for disease outbreak (events=30) from baseline to follow-up (n=269)

	**OR**	**95% CI**	**p-value**	**OR**	**95% CI**	**p-value**
Change in Abdominal pain score from B to FU	0.85	0.73, 0.98	**0.029***	0.89	0.76, 1.04	0.140
Change in Defecation score from B to FU	0.88	0.76, 1.02	0.094			
Change in Social life score from B to FU	0.90	0.79, 1.03	0.133			
Change in Professional life score from B to FU	0.86	0.75, 0.99	**0.030***	0.92	0.78, 1.07	0.265
Change in Sleep score from B to FU	1.00	0.89, 1.13	0.956			
Change in Energy score from B to FU	0.87	0.77, 0.99	**0.042***	0.93	0.79, 1.09	0.355
Change in Anxiety score from B to FU	0.97	0.84, 1.11	0.613			
Change in Self-image score from B to FU	0.89	0.78, 1.03	0.118			
Change in Sexual function score from B to FU	0.94	0.80, 1.09	0.382			
Change in Joint pain score from B to FU	0.89	0.78, 1.03	0.132			
OR: Odds ratio; CI: Confidence intervals; B: Baseline; FU: Follow-up<br>*Statistically significant at level 0.05						

Table S6: Univariate analysis for disease outbreak (events=15) from baseline to follow-up in CD patients (n=199)

	**OR**	**95% CI**	**p-value**
Change in Abdominal pain score from B to FU	0.83	0.69, 0.99	**0.042***
Change in Defecation score from B to FU	0.83	0.68, 1.01	0.061
Change in Social life score from B to FU	0.93	0.78, 1.12	0.433
Change in Professional life score from B to FU	0.87	0.72, 1.04	0.113
Change in Sleep score from B to FU	1.02	0.88, 1.19	0.781
Change in Energy score from B to FU	0.89	0.74, 1.07	0.205
Change in Anxiety score from B to FU	0.95	0.79, 1.14	0.581
Change in Self-image score from B to FU	0.87	0.73, 1.04	0.117
Change in Sexual function score from B to FU	0.89	0.74, 1.06	0.192
Change in Joint pain score from B to FU	0.85	0.70, 1.03	0.094

OR: Odds ratio; CI: Confidence intervals; B: Baseline; FU: Follow-up
*Statistically significant at level 0.05

Table S7: Univariate analysis for disease outbreak (events=15) from baseline to follow-up in UC patients (n=101)

	**OR**	**95% CI**	**p-value**
Change in Abdominal pain score from B to FU	0.84	0.61, 1.16	0.285
Change in Defecation score from B to FU	0.97	0.76, 1.25	0.827
Change in Social life score from B to FU	0.86	0.69, 1.05	0.144
Change in Professional life score from B to FU	0.85	0.69, 1.05	0.135
Change in Sleep score from B to FU	0.97	0.78, 1.22	0.804
Change in Energy score from B to FU	0.88	0.72, 1.07	0.195
Change in Anxiety score from B to FU	1.01	0.81, 1.25	0.962
Change in Self-image score from B to FU	0.96	0.77, 1.19	0.725
Change in Sexual function score from B to FU	0.91	0.72, 1.15	0.443
Change in Joint pain score from B to FU	1.06	0.85, 1.33	0.594

OR: Odds ratio; CI: Confidence intervals; B: Baseline; FU: Follow-up
